# Accurate detection and quantification of seasonal abundance of American bullfrog (*Lithobates catesbeianus*) using ddPCR eDNA assays

**DOI:** 10.1038/s41598-021-90771-w

**Published:** 2021-05-28

**Authors:** Teun Everts, David Halfmaerten, Sabrina Neyrinck, Nico De Regge, Hans Jacquemyn, Rein Brys

**Affiliations:** 1 Research Institute for Nature and Forest, Geraardsbergen, Belgium; 2 Department of Biology, Plant Conservation and Population Biology, KU Leuven, Leuven, Belgium

**Keywords:** Molecular biology, Freshwater ecology, Invasive species, Conservation biology

## Abstract

The invasive American bullfrog (*Lithobates catesbeianus*) imperils freshwater biodiversity worldwide. Effective management hinges on early detection of incipient invasions and subsequent rapid response, as established populations are extremely difficult to eradicate. Although environmental DNA (eDNA) detection methods provide a highly sensitive alternative to conventional surveillance techniques, extensive testing is imperative to generate reliable output. Here, we tested and compared the performance of two primer/probe assays to detect and quantify the abundance of bullfrogs in Western Europe in silico and in situ using digital droplet PCR (ddPCR). Although both assays proved to be equally target-specific and sensitive, one outperformed the other in ddPCR detection resolution (i.e*.*, distinguishing groups of target-positive and target-negative droplets), and hence was selected for further analyses. Mesocosm experiments revealed that tadpole abundance and biomass explained 99% of the variation in eDNA concentration. Because per individual eDNA emission rates did not differ significantly among tadpoles and juveniles, and adults mostly reside out of the water, eDNA concentration can be used as an approximation of local bullfrog abundance in natural populations. Seasonal eDNA patterns in three colonized ponds showed parallel fluctuations in bullfrog eDNA concentration. An increase in eDNA concentration was detected in spring, followed by a strong peak coinciding with the breeding season (August, September or October), and continuously low eDNA concentrations during winter. With this study, we report the validation process required for appropriately implementing eDNA barcoding analyses in lentic systems. We demonstrate that this technique can serve as a solid and reliable tool to detect the early stages of bullfrog invasions and to quantify temporal changes in abundance that will be useful in coordinating large-scale bullfrog eradication programs and evaluating their efficiency.

## Introduction

Freshwater ecosystems are highly biodiverse habitats that contain a large number of distinct animal and plant species, and fulfill important ecosystem functions. Unfortunately, many freshwater ecosystems around the world have been exposed to a vast range of anthropogenic disturbances that jeopardize biodiversity^1–3^. Among these anthropogenic stressors are Alien Invasive Species (AIS)^4^, which are recognized as one of the leading causes of animal extinctions worldwide^5,6^, and as such contribute to the global biological homogenization^7^. Once successfully established, the complete eradication of AIS becomes exceedingly difficult^8^. Next to prevention, early detection of incipient invasions followed by rapid and appropriate eradication actions is thus considered to be the most effective and cost-efficient approach to counter AIS^9,10^. Reliable and accurate monitoring programs that can be rolled out on a large spatial scale are therefore a prerequisite for biosurveillance and the development of successful long-term extermination strategies^11,12^. However, since discovering the early stages of invasion can be challenging via conventional surveillance methods^13^, more sensitive monitoring techniques are imperative to cope with freshwater invaders^14^.

In recent years, environmental DNA (eDNA) barcoding has emerged as a promising non-destructive molecular tool for monitoring aquatic species^15,16^. This technique is based on the detection of DNA traces that are continuously shed or excreted by organisms in their environment, serving as molecular fingerprints^17,18^. An aquatic eDNA survey typically involves the collection of water samples, after which the comprised target DNA, albeit fragmented and highly diluted (< 200 pg/L), is concentrated, amplified, and analyzed^19^. Given the high sensitivity of eDNA detection methods in combination with the short lifespan and constrained spatial dispersal of eDNA in lentic systems, the target species’ presence can be inferred accurately and precisely^18–20^. Indeed, a myriad of studies have demonstrated much higher detection probabilities obtained via eDNA screening than realized with conventional methods^21–23^. Moreover, when elusive species present in low abundances are targeted, eDNA detection methods have been shown to outperform conventional monitoring techniques in handling time and cost-effectiveness, especially in inaccessible habitats^18,24^.

Prior to implementation of large-scale and standardized surveillance programs, eDNA barcoding assays, however, require extensive validation efforts to avoid methodological flaws that may potentially culminate in incorrect outcomes^19,24–26^ and, in turn, in ineffective management interventions^27^. False positives (type I errors), for instance, can emerge from non-specific binding of primers or sample contamination. Alternatively, deficient primer binding efficiency, non-target template competition, sample degradation, poor DNA extraction efficiency or PCR inhibition can culminate in false negatives (type II errors)^19,28–30^. Intensively evaluating the specificity and sensitivity of primer/probe assays in silico, in vitro, and in situ is therefore a crucial, yet frequently undervalued, step to avoid such errors^30–33^.

Apart from serving as a highly sensitive detection tool, eDNA analyses can also provide insights in the abundance of target species when combined with quantitative polymerase chain reaction (qPCR) or digital PCR (dPCR)^34,35^. Depending on the system and species, various studies have documented a positive correlation between the eDNA concentration measured and target species abundance or biomass, both under artificial experimental settings^36–38^ and natural conditions^39,40^. However, to serve as a reliable tool to detect species at very low abundances in the field and to extrapolate eDNA concentrations into abundance estimates, temporal insights in eDNA patterns throughout the year are needed^41,42^. Only then, results generated by eDNA analyses can be appropriately interpreted and adopted to monitor aquatic populations in space and time in a standardized manner^25,33^.

**Figure 1:**
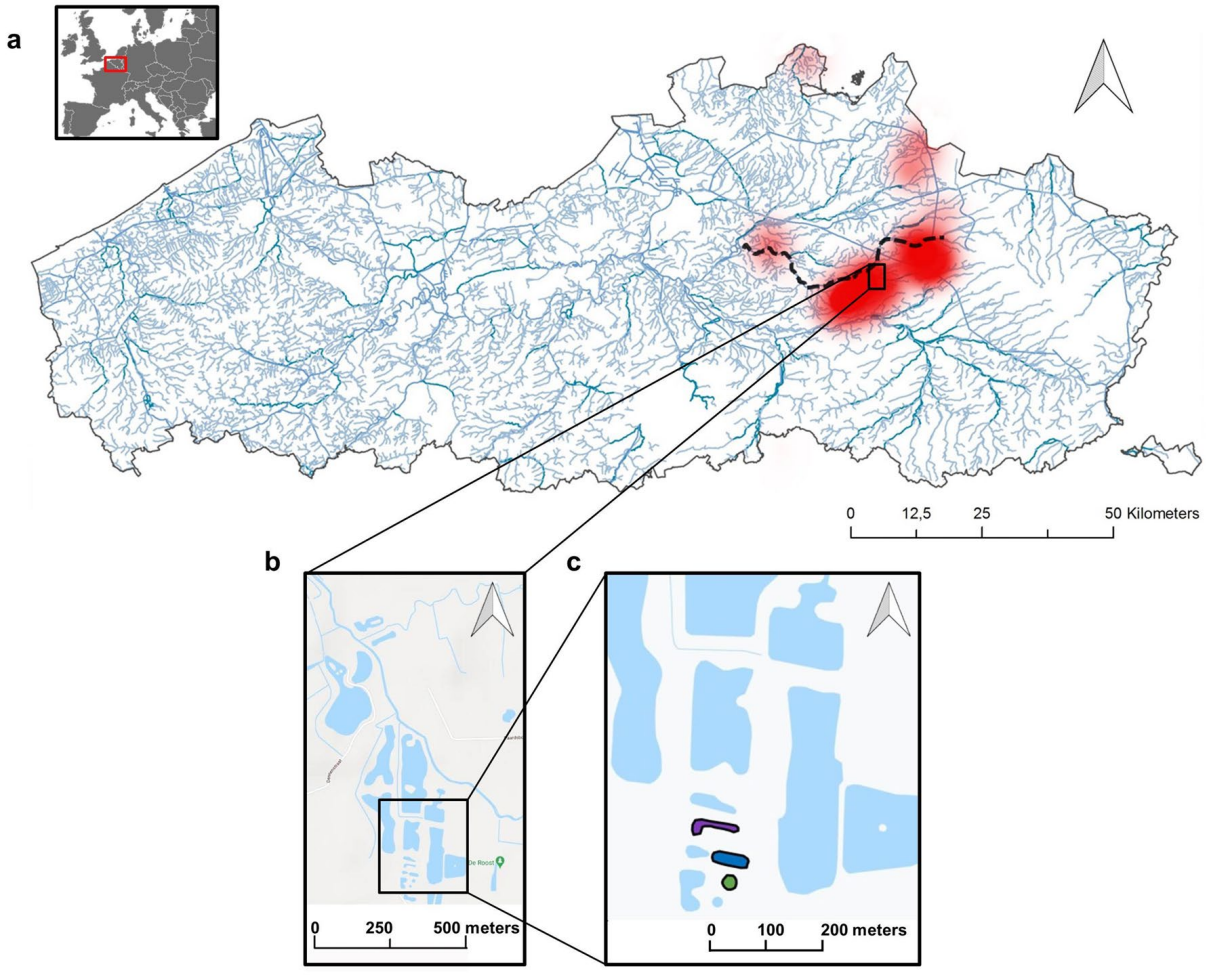
(**a**) A map of Flanders (Northern Region of Belgium, Western Europe) highlighting the contemporary bullfrog invasion in red (data derived from eradication campaigns and the open science data platform “waarnemingen.be”). The Grote Nete river is delineated by the black dotted line. (**b**) Location of nature reserve De Roost, where (**c**) the three permanent ponds were situated that were consecutively sampled for 21 months, highlighted in green (pond 1), blue (pond 2), and purple (pond 3). Maps were created in QGIS version 3.10.10 (https://www.qgis.org/) using map data derived from Google.

The American bullfrog (*Lithobates catesbeianus*; hereafter referred to as bullfrog) is a large amphibian that is native to eastern North America, but is currently invading freshwater ecosystems all around the globe, thereby reducing native biodiversity via competition, predation, and the transmission of novel pathogens^43–45^. The species is ranked as one of the 100 most adverse invaders in the world^46^ and is acknowledged by the European Union as a species of concern (EU Regulation 1143/2014). Eradication of established bullfrog populations is a daunting endeavor to which an increasing amount of resources and efforts are being devoted worldwide, although chances of success remain slim^47–49^. This is reflected in the Belgian bullfrog invasion, which commenced in the 1990s and is, despite eradication efforts, expanding ever-since^50,51^. Apart from a number of satellite populations, the main concern is a well-established large metapopulation spanning an area of roughly 100 km^2^ and covering several hundreds of water bodies along the river Grote Nete (Antwerp) (Fig. 1a). This stream serves as a dispersal corridor for bullfrogs and facilitates the colonisation of the numerous suitable ponds in its immediate vicinity, making active control challenging^51,52^. Disposing of a comprehensive overview of its distribution and local abundance, especially at very low abundances, is an absolute prerequisite for successful control of AIS^23^. However, this is currently one of the main shortcomings of bullfrog eradication campaigns in Belgium.

Targeted eDNA analyses might be the key to successful eradication of not only this invader, but aquatic AIS in general^27^. It has previously been demonstrated that this technique can improve the detection rate and cost-efficiency of bullfrog surveillance substantially^23^ and a handful of studies have already designed different bullfrog-specific primer/probe assays for eDNA-based monitoring^15,31,32^. However, the relative performance of these bullfrog-specific assays remains largely unknown. In addition, no studies have explored the relationship between bullfrog abundance and eDNA concentration, and how this is influenced by bullfrog phenology, which could provide valuable information for the development and evaluation of large-scale eradication strategies.

In this study we aim to (i) determine and compare the specificity and sensitivity of two digital droplet PCR (ddPCR) eDNA primer/probe assays for the detection of bullfrogs in Western Europe, (ii) assess whether eDNA concentrations match bullfrog abundance and/or biomass in a mesocosm experiment, and (iii) investigate seasonal patterns in eDNA profiles in natural populations, in order to demonstrate that eDNA analyses targeting bullfrogs can be implemented as a robust and operational tool for routine use to complement large-scale eradication campaigns.

## Results

### Primer/probe assay validation

**Figure 2:**
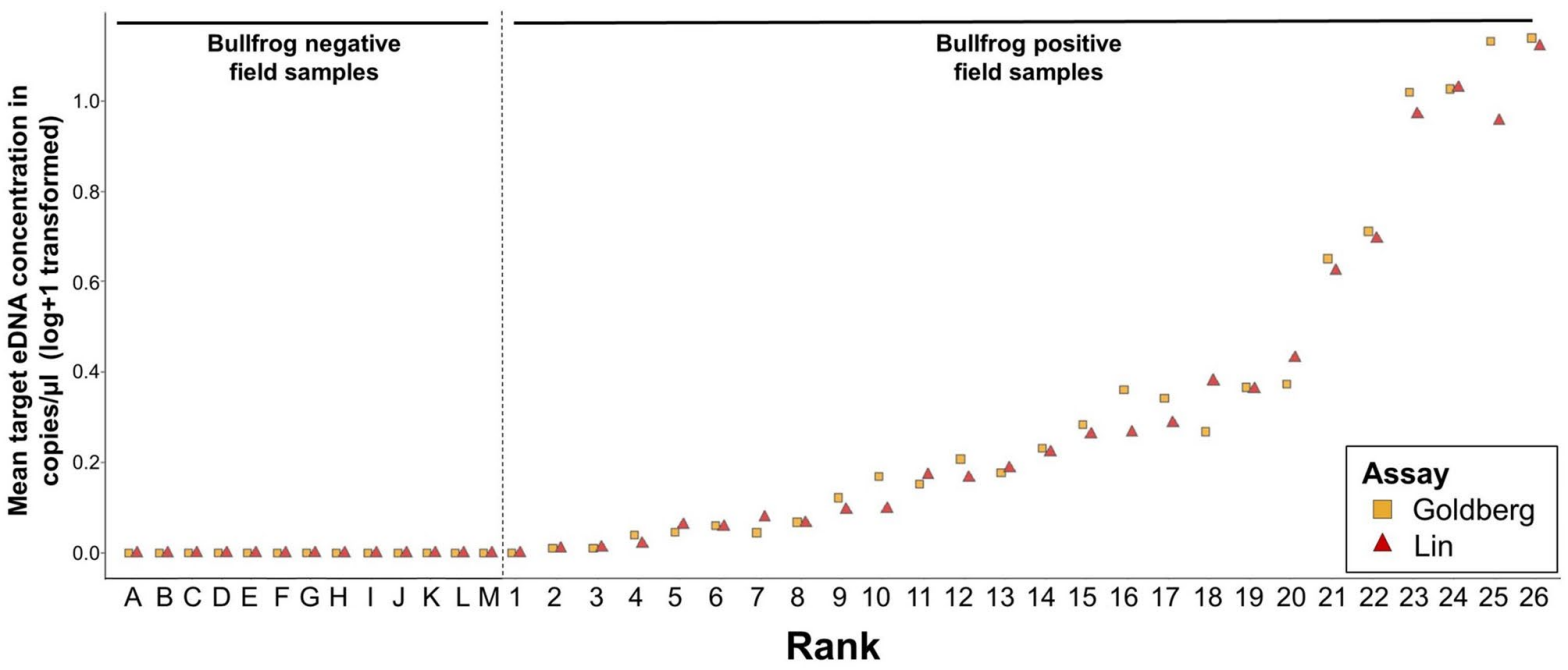
Mean bullfrog eDNA concentrations of field-collected samples quantified with the Goldberg and Lin primer/probes for the in situ evaluation of specificity (ranks A–M) and sensitivity (ranks 1–26). Samples corresponding to ranks A to M were collected from water bodies free from bullfrogs, but harboring the most common co-occurring amphibian species native to Western Europe (see Supplementary Table S1). Ranks 1 (~ 0.025 copies/µL) to 25 (~ 12.45 copies/µL) reflect a ranking of increasing mean bullfrog eDNA concentrations of samples collected from natural water bodies. Rank 26 represents a bullfrog tissue sample functioning as a positive reference in this study. Per sample, the mean log + 1-transformed eDNA concentrations expressed as copies per µL of three technical replicates are plotted as orange squares for the Goldberg and as red triangles for the Lin assay.

**Figure 3:**
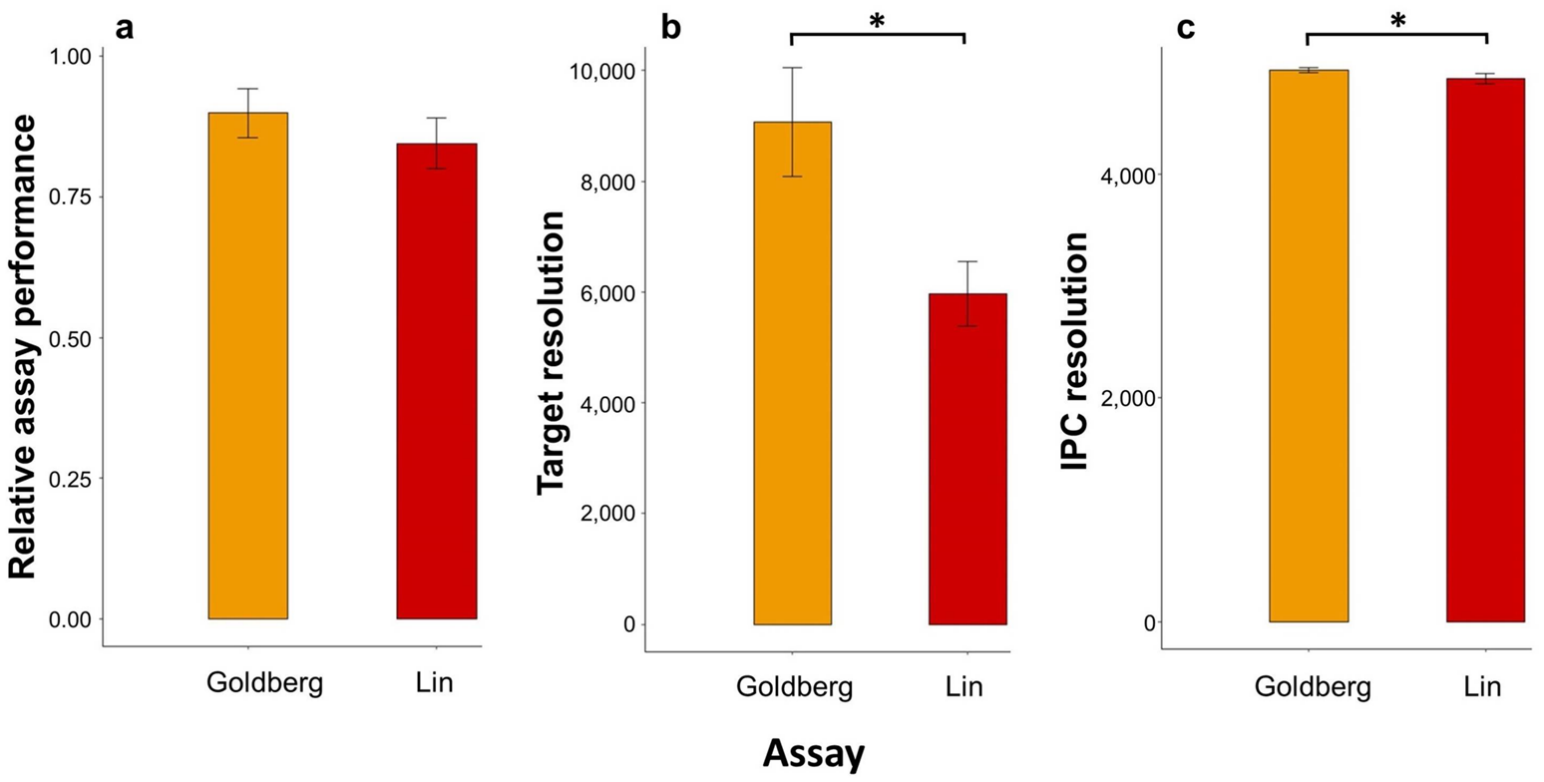
Results of the sensitivity tests of the Goldberg and Lin primer/probe assays using eDNA samples from natural water bodies (corresponding to Ranks 1–25 in Fig. 2). (**a**) Mean (± 1 s.e.m.) relative performance in bullfrog eDNA detection (in terms of eDNA concentration quantified per sample) and the mean (± 1 s.e.m.) (**b**) target and (**c**) IPC detection resolution. The detection resolution was calculated as the difference in fluorescence between positive and negative ddPCR droplets.

The in silico specificity analyses of both primer/probe assays showed multiple mismatches in the binding regions of the corresponding bullfrog sequence with most Western European amphibian species that are likely to co-occur with bullfrogs (Supplementary Fig. S1). Each of the field samples (*n* = 13) taken from water bodies harboring the most common amphibian species indigenous to Western Europe, but no bullfrogs (Supplementary Table S1), showed no bullfrog eDNA amplification for either assay (Fig. 2). The droplet generation during preparatory ddPCR steps in the 25 bullfrog positive field samples and the positive reference sample containing bullfrog tissue resulted on average in 16,119 ± 95 (s.e.m.) accepted droplets, ranging between 8686 and 19,450 droplets. The mean relative performance of the Goldberg and Lin assay on bullfrog positive field samples (in terms of target eDNA concentration quantified per sample) was 0.90 ± 0.043 and 0.84 ± 0.045 respectively, and did not significantly differ between both assays (*t*_25_ = 1.19, *P* = 0.24) (Fig. 3a). The mean target resolution of the Goldberg assay (9065.53 ± 982.32) was significantly greater (*V*_25_ = 3.00, *P* < 0.001) than that of the Lin assay (5968.10 ± 589.7) (Fig. 3b), which was also the case for the mean resolution of the internal positive control (IPC) (*V*_25_ = 0.01, *P* < 0.001), resulting in a mean IPC resolution of 4928.31 ± 21.9 and 4855.77 ± 43.98 for the Goldberg and Lin assay, respectively (Fig. 3c). No positive target detection was obtained from each of the technical negative controls included in the workflow, while all positive reference samples were successfully amplified.

### Mesocosm experiment

**Figure 4:**
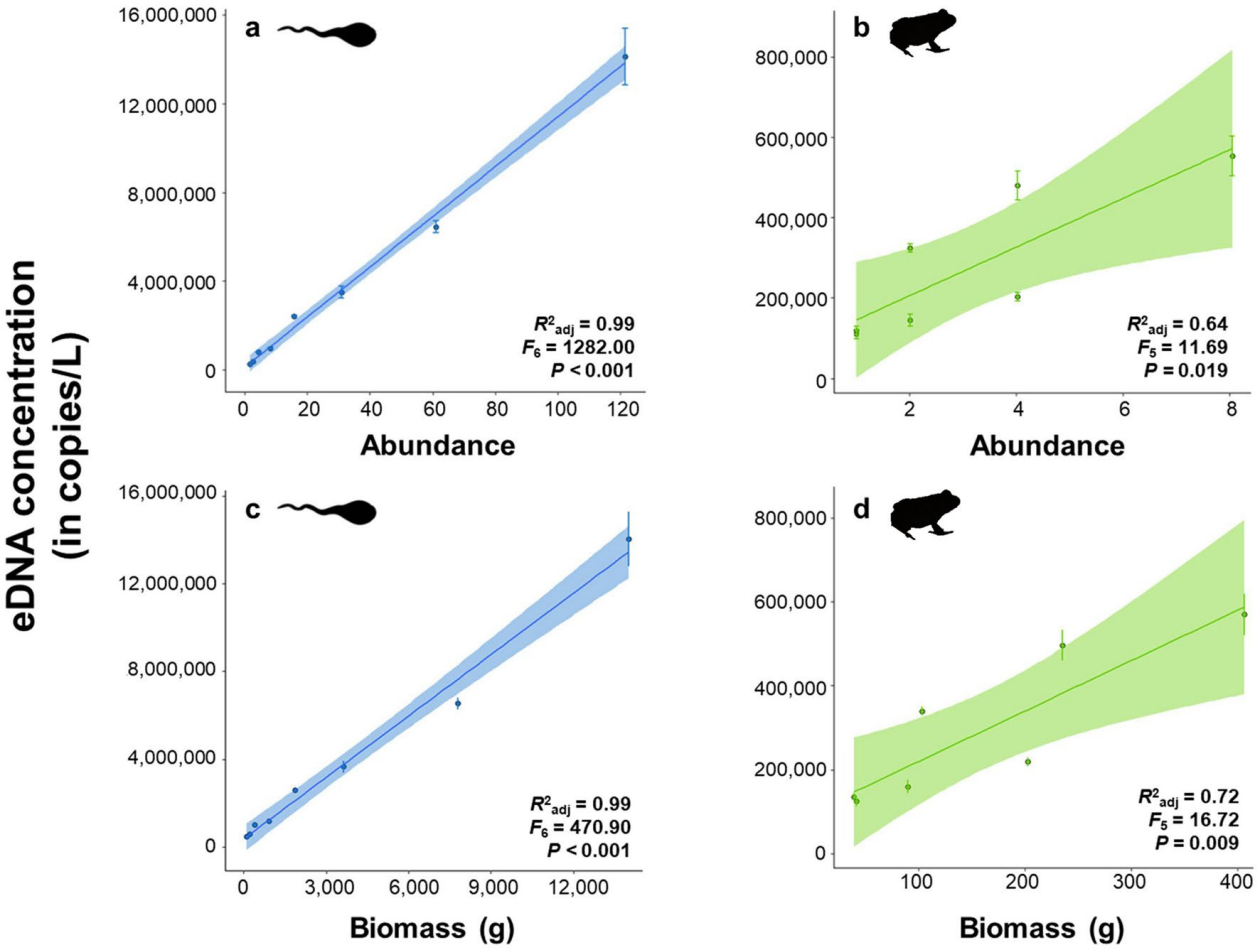
The relation between bullfrog eDNA concentration and abundance (**a**,**b**) and total biomass (**c**,**d**) of tadpoles (blue) and juveniles (green) stocked in the mesocosms, expressed as copies per liter filtered water. Dots represent the means of the technical replicates with the shaded areas around the regression line indicating the 95% confidence intervals. *R*^2^_adj_ values, effect sizes, and *P* values of each regression are given in the bottom right corner of the corresponding graph.

**Figure 5:**
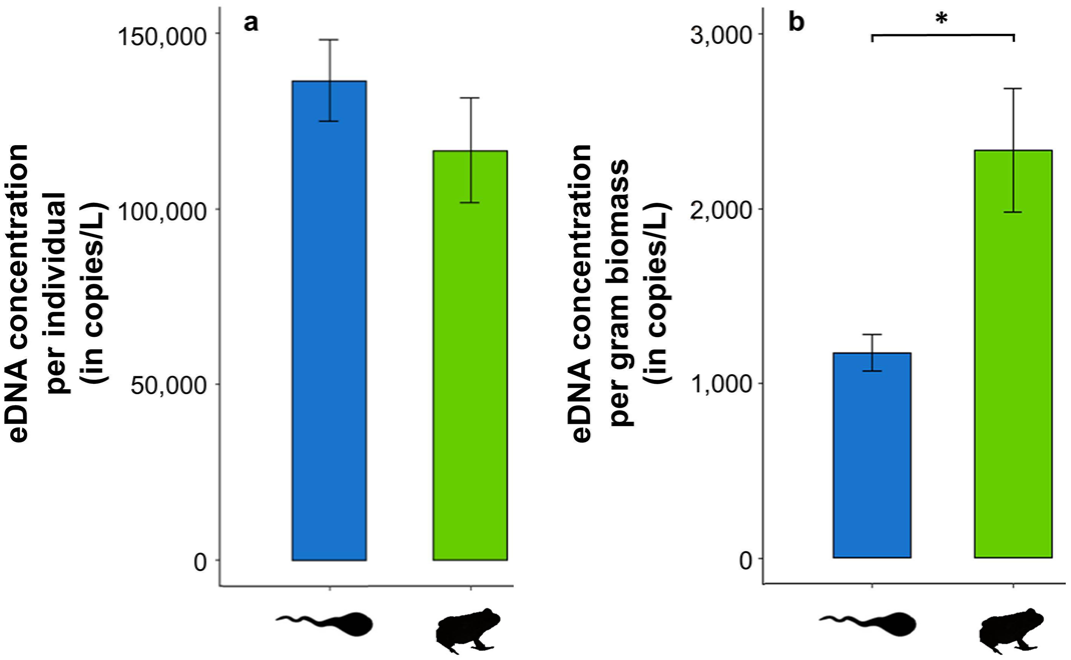
Average (± 1 s.e.m.) per individual (**a**) and per gram biomass (**b**) eDNA concentration of bullfrog tadpoles (blue) and juveniles (green) calculated across mesocosms and expressed as copies per liter filtered water.

No bullfrog eDNA was detected in the water used for the mesocosm experiment prior to the introduction of bullfrogs, nor from the technical negative ddPCR controls included in the workflow. Positive reference samples were successfully amplified. Although the mean number of accepted generated droplets was 15,287 ± 167, three measurements fell below the threshold of 8000 generated droplets, and were consequently discarded. The target eDNA concentration in the mesocosms significantly increased with the abundance of bullfrog tadpoles and juveniles (tadpoles: *F*_6_ = 1282.00, *R*^2^_adj_ = 0.99, *P* < 0.001; juveniles: *F*_5_ = 11.69, *R*^2^_adj_ = 0.64 ; *P* = 0.019) (Fig. 4a,b) and total biomass (tadpoles: *F*_6_ = 470.90, *R*^2^_adj_ = 0.99, *P* < 0.001; juveniles: *F*_5_ = 16.72, *R*^2^_adj_ = 0.72, *P* = 0.009) (Fig. 4c,d). The number of bullfrog eDNA copies per liter filtered water ranged from 180,014 ± 3841 copies for the lowest tadpole abundance (i.e*.*, one tadpole) up to 14,134,307 ± 1,278,879 copies for the highest abundance (i.e*.*, 121 tadpoles), and between 143,307 ± 10,912 copies for the lowest juvenile abundance (i.e*.*, one juvenile) up to 548,331 ± 45,163 copies for the highest abundance (i.e*.*, 8 juveniles). Across all mesocosms, the average per individual eDNA emission rate from tadpoles did not significantly differ from that of the juveniles (*W* = 16.00, *P* = 0.19) (Fig. 5a), whereas average tadpole eDNA emission rates per unit of biomass were significantly lower relative to juvenile bullfrogs (*W* = 49.00, *P* = 0.014) (Fig. 5b).

### Seasonal eDNA patterns

**Figure 6:**
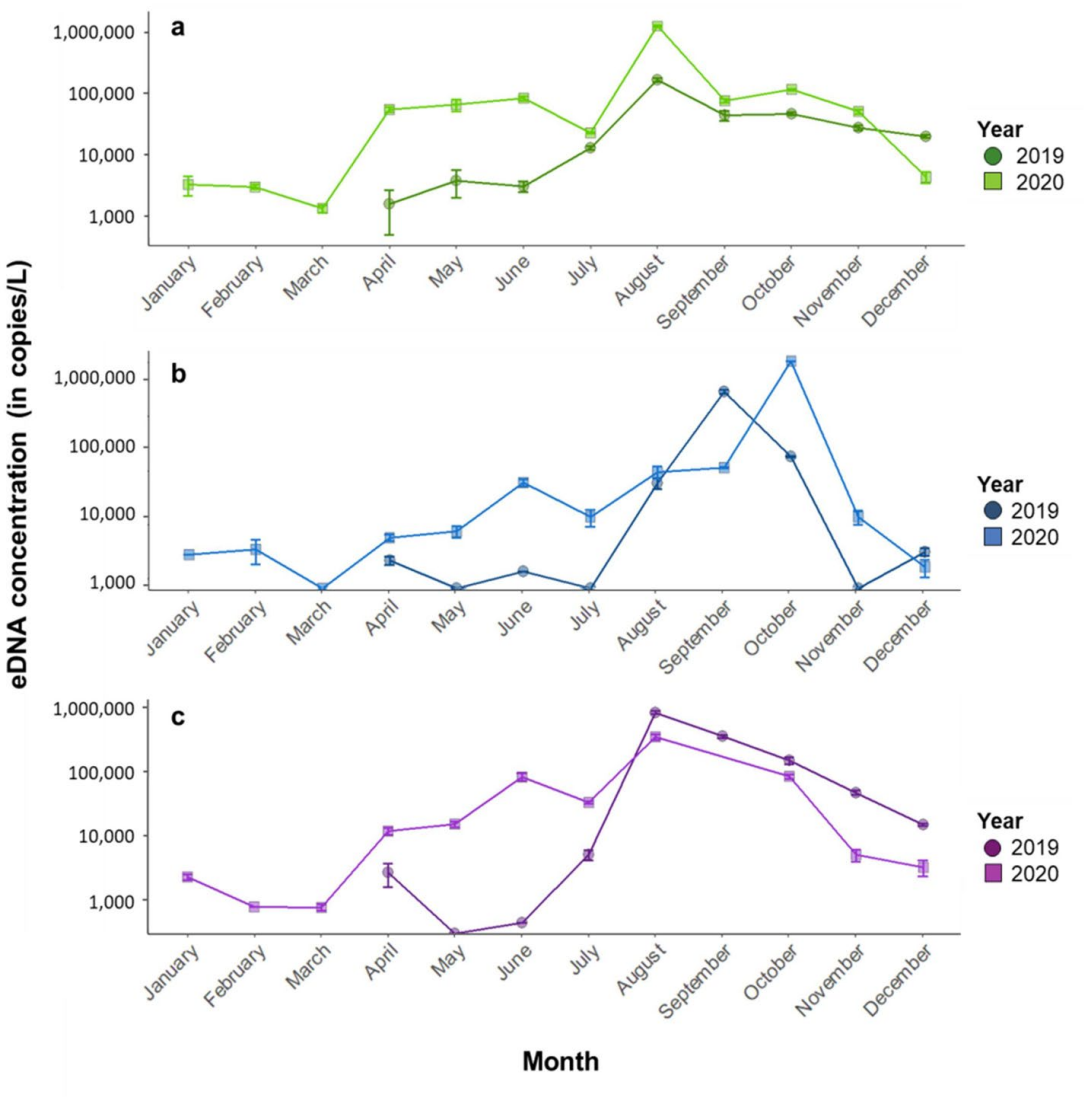
Monthly bullfrog eDNA concentrations on a logarithmic scale, expressed as the average (± 1 s.e.m.) number of copies per liter filtered water of three technical replicates per filter in three natural ponds known to be heavily infested by bullfrog, located at the core of the Belgian metapopulation (see Fig. 1): pond 1 (**a**), pond 2 (**b**), and pond 3 (**c**). Samples collected in 2019 and 2020 are represented by circles and squares, respectively. Pond 3 was not sampled in September 2020 as it had completely dried out. The intersection of the x-axis and y-axis corresponds to an eDNA concentration of 0.

Across all seasonal samples analyzed, the total number of generated droplets was on average 15,935 ± 75, with a minimum of 8511 droplets. Over the entire survey period (21 months), the mean bullfrog eDNA concentration (expressed as copies per liter filtered water) was highest in pond 2 (125,468 ± 3919) and lowest in pond 3 (87,627 ± 6673), with intermediate concentrations in pond 1 (107,974 ± 6293). Bullfrog eDNA concentrations increased in spring and reached distinct peaks in August for pond 1 (181,506 ± 13,699 and 1,395,273 ± 52,099 copies per liter water in 2019 and 2020 respectively) and pond 3 (724,024 ± 43,765 and 303,215 ± 28,518 copies per liter water in 2019 and 2020 respectively), and in September–October for pond 2 (630,233 ± 44,462 and 1,739,803 ± 8332 copies per liter water in 2019 and 2020 respectively) (Fig. 6a–c). Bullfrog eDNA concentrations gradually decreased following these peaks, and reached the lowest concentrations from November to March for all three ponds during both years (on average 9777 ± 1032 copies per liter water). Although bullfrogs were detected year-round in pond 1, no bullfrog eDNA was detected in pond 2 in May, July, and November in 2019 and in March 2020, whereas in pond 3 bullfrogs were not detected in May 2019. Negative controls retrieved no bullfrog eDNA copies, whereas all positive reference controls were successfully amplified.

## Discussion

Since eDNA detection methods are cost-effective, non-invasive, applicable on a large spatial scale, and extremely sensitive, they hold great potential for supporting the management of aquatic AIS^19,23,27,49^. Formally validating primer/probe assays for an explicitly defined geographical study area must be the first step in the development of large-scale eDNA-based surveillance protocols to ensure that the generated findings are reliable for the resulting decisions to be pertinent^26–30^. One of the most important aspects that has to be considered when evaluating the performance of a primer/probe assay is its specificity, i.e*.*, the degree to which it specifically amplifies and detects the target sequence without interacting with genetic material of co-occurring non-target species^31,32,53^. Specificity evaluation is especially important in the context of aquatic eDNA screening, as its highly degraded nature necessitates the use of shorter sequences as barcodes, which inherently harbor fewer species-specific regions^19,29^. Primer specificity of the bullfrog-specific Goldberg and Lin assays was previously excessively evaluated in silico for potential cross-amplification with co-occurring non-target species inhabiting the Western US and the Beijing area, respectively^31,32,53^. However, because species-specific primers are developed for specific geographical areas and their performance can vary geographically, they cannot be used blindly across regions^31,32^. A recent study, for instance, demonstrated that a primer pair that specifically targeted bullfrogs in France co-amplified non-target species eDNA when used in China^32^. Because false positive detections could lead to unnecessary costly management interventions when combatting AIS^27^, it is imperative to carry out additional specificity tests for non-target species inhabiting a particular study area. The in silico evaluation performed here showed that the targeted regions of both assays included many nucleotide mismatches between bullfrogs and Western European amphibian species, which indicates very little potential for cross-amplification and hence confirmed their specificity (Supplementary Fig. S1)^28^.

Nevertheless, in vitro and in situ tests should complement in silico specificity evaluation, since primers can interact with non-target sequences regardless of nucleotide mismatches^28,54^ and the outcome of DNA amplification can also depend on sample and system specific PCR conditions^55^. Although primer specificity was already comprehensively evaluated in vitro^31,32,53^, we conducted additional in situ tests on natural eDNA samples collected from water bodies located outside the distribution area of bullfrogs in Belgium containing eDNA from a mixture of indigenous amphibian species under variable environmental conditions (Supplementary Table S1). In this way, a more reliable indication of an assay’s specificity in the geographic area of interest is obtained compared to an in vitro approach^30^. When both assays were run on water samples originating from bullfrog-free water bodies harboring most amphibian species indigenous to Western Europe that co-occur with bullfrogs, neither of the two assays under study showed eDNA amplification (Fig. 2), while the IPC and the bullfrog positive reference samples were successfully amplified. Therefore, it can be concluded that both assays specifically target and detect bullfrog eDNA in Western European water bodies.

A second aspect that determines primer/probe assay performance is its sensitivity, i.e*.*, its ability to detect even the slightest traces of target eDNA. Highly sensitive assays enable the detection of aquatic AIS at very low abundances and hence are key to cost-effective management^9,10,27,28^. The sensitivity of primer/probe assays is typically quantified using a dilution series of target DNA from tissue extracts to determine the lowest detectable DNA concentration, which was previously carried out for the Goldberg assay and demonstrated its high sensitivity^31^. However, such tests are conducted under optimal conditions and therefore poorly reflect their true sensitivity under natural conditions, where abundant non-target eDNA and environmental factors impede target eDNA amplification to varying extents^25,30,56^. Therefore, we tested the performance of both primer/probe assays on a range of natural eDNA samples collected from water bodies that varied widely in ecological conditions and were invaded by bullfrogs with different intensities. The results showed that the two assays did not significantly differ in the concentration of bullfrog eDNA quantified per sample (Figs. 2, 3a). However, the Goldberg assay outperformed the Lin assay in terms of ddPCR detection resolution (Fig. 3b,c), which is defined as an assay’s ability to separate groups of target-positive and target-negative droplets and is jointly determined by primer/probe binding efficiency, partial PCR inhibition, and delayed PCR onset^57,58^. Because the stagnant nature of lentic waters often results in the build-up of PCR inhibiting compounds^59^, ddPCR detection resolution is a third important feature of primer/probe assays that needs to be taken into account. A higher detection resolution reduces the probability that inhibited PCR amplifications fall below the fluorescent threshold for positive droplets^57^. Therefore, the Goldberg assay is more resilient to false negative amplifications, and hence is a more reliable indicator of bullfrog presence and abundance in natural conditions compared to the Lin assay, especially in early invasion stages and turbid waters. The higher detection resolution of the IPC assay when ran in duplex with the Goldberg assay relative to when duplexed with the Lin assay was, at least partly, expected, as the optimal annealing temperature of the primers and probes targeting the IPC is equal to that of the Goldberg assay (i.e*.*, 60 °C), and differs from the Lin assay (i.e*.*, 55 °C). Nevertheless, this implies that the correction factor based on IPC measurements, and thus final eDNA concentrations, is more accurate when adopting the Goldberg rather than the Lin assay when combined with the particular IPC used in this work. Altogether, our results indicate that for the ddPCR approach applied, the Goldberg assay is more robust in detecting and quantifying bullfrog eDNA in Western Europe compared to the Lin assay, which led us to select this assay for further analyses.

The controlled mesocosm experiment showed that the Goldberg assay quantified eDNA according to bullfrog abundance and biomass. Especially at the tadpole life stage, abundance predicted the obtained eDNA concentrations remarkably well (explaining 99% of the variance as indicated by *R*^2^_adj_) (Fig. 4a). This strong correlation was well above the in vitro average of 82% reported in a recent meta-analysis^34^, suggesting relatively homogenous DNA discharge rates among bullfrog tadpoles and densities compared to other species. Moreover, eDNA quantification via ddPCR has been shown to be more precise than via qPCR^35–37,60^, the latter being overrepresented in the meta-analysis^34^, and can additionally explain the higher predictive power observed, especially in combination with the inclusion of IPC’s in our workflow^30^. Although significant, this relation was less pronounced for bullfrog juveniles (Fig. 4b). Because the gills of tadpoles are functionally exchanged for lungs during metamorphosis^61^, juvenile bullfrogs are not permanently submerged in the water, but frequently reside on aquatic vegetation or on land near water bodies. Since our mesocosms were equipped with terrestrial islands for juveniles to dwell out of the water, this might have introduced more variation in the association between eDNA concentration and juvenile bullfrog abundance. Nevertheless, the average per individual eDNA emission rate did not differ significantly between bullfrog tadpoles and juveniles across mesocosms (Fig. 5a). This is a prerequisite for the application of eDNA-based estimations of bullfrog population sizes in natural conditions because bullfrog tadpoles metamorphose in their second year of life and thus co-occur with conspecific juveniles^62^. At least under controlled conditions, the Goldberg ddPCR primer/probe assay thus provides an accurate prediction of bullfrog abundance in the water column irrespective of both life stages. Alternatively, the per gram eDNA emission rate was significantly higher for the lighter juveniles relative to the heavier tadpoles (Fig. 5b). Tadpoles are heavier than juveniles because accumulated food reserves are depleted during metamorphosis^61^. This pattern also emerged in other studies such as with bluegill sunfish (*Lepomis macrochirus*), where heavier adults on average had a higher per individual eDNA release rate, whereas their per biomass eDNA release rate was lower relative to the lighter juveniles^63^. This finding, in conjunction with the highly variable biomass distribution among bullfrog individuals and life stages, makes it more appropriate to estimate bullfrog abundance rather than biomass from eDNA signals picked up in blind systems.

Under field conditions, however, relationships between abundance and eDNA concentration can be affected by other factors that introduce additional variation^18,19,24^. It was shown that species abundance explained on average a lower percentage of the variation in eDNA concentrations under natural than under artificial conditions (57% and 82% respectively)^34^. This can be attributed to the complex interplay of several factors affecting eDNA production and degradation rates, such as temperature, UV exposure, pH, microbial activity^53,64,65^, animal behavior^40^, and season- and age-dependent eDNA shredding rates^41,63^. In addition, the accumulation of particulate and dissolved substances (such as calcium ions, humic and tannic acids, polymers, etc.) and eDNA from non-target species can further hamper target eDNA amplification^56,59^, resulting in underestimations of the actual abundance of target organisms. However, the amplification in ddPCR is less susceptible to inhibitory substances than qPCR, and is therefore more robust, especially when the target DNA template is scarce^35–38,57–60^. Furthermore, the inclusion of IPCs in our workflow not only enables a reliable differentiation of true negatives from PCR errors, but also contributes to the standardization of variation in eDNA concentrations resulting from sample-specific suboptimal DNA extraction or partial ddPCR inhibition^30,38,66^. This indicates that bullfrog eDNA concentrations quantified with this protocol can be used as rough estimates of the colonization status of natural lentic systems given an integrated and standardized sampling design^34,59^ and hence provide valuable quantitative information for conservation managers.

Since eDNA patterns in the field are expected to be influenced by seasonal alterations in both the ecology of the target species and its environment^40,64,65^, we assessed year-round temporal variation in bullfrog eDNA concentrations for two subsequent years in three natural ponds. Over this 21 month study period, the three ponds showed similar patterns in seasonal variation in bullfrog eDNA concentrations: a consistently low concentration from November to March preceded a small increase during spring (between March and July), followed by a large peak in late summer (between August and October) (Fig. 6). These temporal eDNA patterns largely reflect the seasonal phenology of bullfrog populations in Western Europe. Mating peaks during late spring and early summer and a new generation of tadpoles emerges between July and September^67^. Reproduction in amphibian species has been documented to result in two peaks in eDNA concentrations in natural conditions, one representing the reproductive behavior of the adults and the subsequent mass release of gametes, followed by a second peak representing the emergence of tadpoles^41,68,69^. Since we sampled our study ponds on a monthly basis whereas the timing between bullfrog breeding and tadpole emergence ranges between a few days up to 1 week^62,70^, it is plausible that we could not differentiate these two peaks. Moreover, given that the breeding season of bullfrogs spans a few months, and that multiple breeding events can occur in the same water body^62,70^, mating and emergence of bullfrog tadpoles presumably cannot be distinguished in terms of eDNA concentration. Alternatively, the studied ponds may have served as refuges for first-year juveniles escaping competition and predation from congener adults in nearby breeding ponds, or as stepping stones or foraging sites^47,51,62,70,71^. Because bullfrog tadpoles require at least 1 year of permanent water to survive until metamorphosis^47,62^, this hypothesis was supported by the complete desiccation of one study pond for 1 month, combined with the observation of a large number of bullfrog juveniles in each study pond in the summer of both years. In any case, even though adult abundance is a strong determinant for population growth rate^72,73^, it is expected to have only a minor influence on the observed peaks in eDNA concentrations, since adults are typically outnumbered by tadpoles and juveniles while mostly residing out of the water^23,51,70^. It should also be noted that the observed peaks in eDNA concentrations could have been intensified by the dry summers of 2019 and 2020, which resulted in declines of the water table.

As temperatures decrease in autumn and cross the threshold of 15 °C, which generally occurs from October to March, bullfrogs enter winter torpor. They mostly hibernate semi-immersed in sediment at the bottom of a pond, but winter lethargy or adopting terrestrial hibernacula instead is not exceptional^52,67^. Therefore, the reduced metabolic rate, and hence eDNA release, during hibernation^39^, or the lowered abundance of juveniles in water bodies during winter could explain the observed decrease in measured eDNA concentrations following the peak in late summer. Alternatively, because bullfrogs produce clutches that contain up to 30,000 eggs of which the majority hatch^47,51,70^, the extremely low eDNA signals throughout the winter suggest the absence of tadpoles and hence breeding in these study ponds, and thus support the hypothesis that the observed temporal patterns mainly originated from bullfrog juveniles. In any case, these findings reliably indicate bullfrog presence throughout the winter season, as bullfrog eDNA was previously reported to persist up to 1 week in the water column^20^. In contrast, previous research was unsuccessful in detecting eDNA of two hibernating endangered frog species in headwater streams during the winter season^42^. This suggests that our intense sampling strategy and efficient primer/probe assay could detect even minute quantities of eDNA during the period bullfrogs are inactive or scarcely present. Altogether, these findings confirm previous research demonstrating that eDNA concentrations closely track the seasonal phenology of the target species, and indicate that eDNA signals obtained from summer sampling most accurately approximate local bullfrog abundance. The patterns observed here could thus provide crucial information on the timing when large-scale eDNA detection campaigns and eradication programs targeting the bullfrog would be most fruitful^39,42,69^. Additionally, these results demonstrate that this primer/probe assay is not only able to estimate the colonization status of natural water bodies, but also to track changes in bullfrog abundance, and hence can be used to evaluate the effectiveness of management interventions^49,74^.

With this work, we report the validation process and exploratory research required for appropriately implementing species-specific eDNA analyses using ddPCR technology for reliable detection and quantification in large-scale monitoring campaigns. It was previously demonstrated that aquatic eDNA surveys are more sensitive and less costly than their conventional counterparts, since eDNA samples can be collected in a short period of time and can be processed in bulk^21–23^. Additionally, ddPCR analyses are known to exceed qPCR in terms of cost-effectiveness and eDNA detection and quantification performance, especially at low target species abundance and in turbid waters rich in inhibitory compounds^35–38,60^. We tested two primer/probe assays specifically designed for detecting bullfrogs, and showed that one outperformed the other in detection resolution, which is an imperative feature for the early detection of this invasive species in lentic systems, and hence for rapidly responding with the necessary eradication measures. The mesocosm experiments showed that the most robust ddPCR primer/probe assay can provide a pragmatic approximation of bullfrog abundance independent of life stage, whereas under natural conditions clear seasonal eDNA patterns were revealed in some permanently colonized natural ponds. The outcome of our validation process suggests that this protocol is ready to be implemented in large-scale monitoring campaigns in Western Europe, in order to coordinate, evaluate, and eventually fine-tune such eradication programs^27,49,74^.

## Methods

### Primer/probe assay validation

In the first step, we tested the performance of available primer/probe assays specifically designed for bullfrog eDNA detection, both in terms of specificity and sensitivity. To the best of our knowledge, three primer pairs targeting bullfrog eDNA were published at the onset of this study, which are referred to as the Ficetola^15^, Strickler^53^, and Lin^32^ assays. The lack of a corresponding probe for the Ficetola assay prevents target-specific eDNA quantification and limits its specificity^28,30^, which led to its exclusion. In contrast, three probes of different length were available for the Strickler primers: the original 17 bp MGB probe^53^ and two longer BHQ probes: an adapted 21 bp version^31^ and an unpublished 27 bp version developed by C. S. Goldberg (personal communications) (Table 1). Of the three aforementioned primer/probe combinations, we chose to further test the C. S. Goldberg assay (referred to as the Goldberg assay), since it was characterized by the longest probe (and thus the highest expected specificity^28^) and an annealing temperature identical to the conditions needed for optimal co-amplification of the internal positive control used for quality testing (see section “Laboratory protocol”). The specificity and sensitivity of this Goldberg assay was compared to the assay designed by Lin et al*.*^32^ (referred to as the Lin assay), which was already intensively screened and found to be very robust.

**Table 1:** Sequences of all published primers and probes targeting bullfrog DNA at the mitochondrial cytochrome *b* gene (Ficetola^15^, Strickler^53^/Veldhoen^31^/Goldberg) and at the 16S rRNA gene (Lin^32^), as well as for the internal positive control (IPC) being a 149 bp plasmid insert sequence from the Dengue virus type 2 (INSDC M29095.1).

Target	Assay	Primer/probe	Sequence (5′–3′)
*L. catesbeianus*	Ficetola^15^	LCcytb Forward	TGCCAACGGAGCATCATTC
		LCcytb Reverse	ATAAAGGTAGGAGCCGTAGT
*L. catesbeianus*	Strickler^53^	BullfrogF	TTTTCACTTCATCCTCCCGTTT
	Strickler^53^	BullfrogR	GGGTTGGATGAGCCAGTTTG
	Strickler^53^	Bullfrog MGB Probe	(NED)TTATCGCAGCAGCAAGT(MGB)
	Veldhoen^31^	Bullfrog BHQ Probe	(6FAM)TTATCGCAGCAGCAAGTATGA(ZEN/IBFQ)
	C. S. Goldberg pers. comm	Bullfrog BHQ Probe	(6FAM)TTATCGCAGCAGCAAGTATGATCCACC(ZEN/IBFQ)
*L. catesbeianus*	Lin^32^	qLC16S Forward	GCAGAGATAACCTCTCGT
		qLC16S Reverse	GTCCCATAGGACTGTTCT
		qLC16S BHQ Probe	(6FAM)TGCCCTCCCGAAACTAAGTGAGC(ZEN/IBFQ)
IPC	NEN 6254, 2012	IPC-D2-F	ATGACAGCCACTCCTCCG
		IPC-D2-R	GGAACGAACCAAACAGTCTTC
		IPC-D2-Probe	(HEX)AGCAGAGACCCATTCCCTCAGAGC(ZEN/IBFQ)

The Goldberg assay targets a 84 bp fragment of the mitochondrial cytochrome *b* gene and was specifically designed for the detection of bullfrogs in the Western US, whereas the Lin assay was designed for bullfrog detection in China, targeting a 120 bp fragment of the 16S rRNA gene (Table 1). We assessed the specificity of both assays to detect bullfrogs in Western Europe in a two-step process. First, in silico specificity evaluation was carried out for both assays using available sequences of the most commonly co-occurring Western European amphibian species (*n* = 21) from the International Nucleotide Sequence Database Collaboration (INSDC). The most common sequence for each species was used to visualize mismatches with the corresponding bullfrog sequence using Geneious (version 10.2.6) (Supplementary Fig. S1). Next, in situ specificity tests were performed for both assays by running them on eDNA samples originating from ponds and lakes in Belgium that are known to be free of bullfrogs and to harbor the most common co-occurring indigenous amphibian species (*n* = 13). The native amphibian communities were determined via metabarcoding analyses using the generalist vertebrate specific Riaz primer set^75^, which simultaneously confirmed bullfrog absence (unpublished data, see Supplementary Table S1).

To investigate the sensitivity of both assays in natural conditions, we selected 25 eDNA samples collected from a wide variety of ponds spread over the entire distribution range and that were invaded by bullfrogs, to obtain a gradient in natural bullfrog eDNA concentrations. Both primer/probe assays were then run on each of these samples under optimal PCR conditions in triplicate via ddPCR (see Supplementary Methods). The relative performance in terms of bullfrog DNA concentration retrieved and ddPCR detection resolution (i.e*.*, the difference in mean amplitude of groups of positive and negative droplets^58^) was calculated for every sample along this gradient and contrasted among both assays.

### Mesocosm experiment

To assess the extent to which eDNA concentrations can be related to the abundance and biomass of bullfrogs, a controlled mesocosm experiment was set up at the INBO facility in Linkebeek (Belgium). Sixteen outdoor polystyrene tanks (58 × 54 × 89 cm, volume: 200 L) were filled with 122.5 ± 2.58 L rainwater that was tested for potential bullfrog contamination prior to introducing the animals by filtering 3 L over a similar filter as used in the mesocosm and temporal experiments (see below). Eight of these tanks were dedicated to bullfrog tadpoles (248 individuals in total), and seven tanks to juveniles (22 individuals in total). The tadpoles were partitioned over the mesocosms as follows: 1, 2, 4, 8, 16, 32, 64, and 121 individuals. Given the limited number of juvenile bullfrogs that could be obtained, the density range for this life stage was downscaled as follows: two mesocosms were stocked with one, two, and four juveniles, and one tank was stocked with eight juveniles. The per individual wet weight was measured (tadpoles: 116.9 ± 11.3 g, juveniles: 50.5 ± 7.0 g), and the total wet biomass per mesocosm was calculated. Tanks dedicated to juvenile individuals contained one small artificial, similar sized island to prevent juveniles from drowning. Water samples for eDNA analyses were collected 3 days after introduction of the tadpoles or juveniles to the tanks. From each experimental tank, three 1 L water samples were collected and pooled into one merged water sample. In a next step, 1 L of each of these merged water samples was filtered on a 50 mm diameter syringe disk filter with an integrated 5 µm glass fiber prefilter and a 0.8 µm PES membrane (NatureMetrics, Surrey, England) using a Vampire sampler pump (Buerkle, Bad Bellingen, Germany) with disposable silicone tubing. To minimize the likelihood of cross-contamination, sampling was performed in a low-to-high density direction using sterile disposable material. After filtration, the remaining water inside capsules was expelled by forcing air through the capsule. A total of 16 filters (15 filters from the mesocosms and one control filter) were capped at both ends, and stored at − 21 °C in anticipation of further analyses in the laboratory.

### Seasonal eDNA patterns

Three turbid lentic water bodies between 1 and 1.5 m deep with emergent and open riparian vegetation and thus representative for bullfrog infested water bodies in Belgium^51^, were selected to conduct a long-term temporal sampling survey. These ponds had a surface area of approximately 300 m^2^, differed in shape, and were simultaneously sampled each month between April 2019 and December 2020 for a total of 21 monthly sampling rounds. No sample was collected from pond 3 in September 2020 as it had completely dried out. The ponds were located at the core of the Belgian bullfrog metapopulation (Fig. 1) to guarantee that temporal eDNA patterns closely reflected the natural seasonal alterations in bullfrog abundance and activity. As eDNA is highly diluted and often patchy distributed in the water column^20^, an integrated sample strategy was employed to maximize the total habitat coverage and as such to approach the true eDNA concentration as close as possible^59,76,77^. Therefore, ten to thirty 0.5 L subsamples were collected at five meter intervals around each pond and were pooled to obtain a single integrated, homogenous water sample. Using a long sampling pole with a sterile Whirl–Pak bag (Sigma-Aldrich, Overijse, Belgium) attached at the end, water was sampled from just below the water surface (± 10 cm) as this appears the best section of the water column for eDNA detection in lentic systems^76^. The merged water sample was then filtered using the same filter type as in the mesocosm experiment until the filter was saturated. The volume of filtered water was quantified and recorded for further calculation of target eDNA concentration per liter filtered water. Cross-contamination was impeded by wearing sterile nitrile gloves and using sterile sampling bags, which were discarded after each pond was sampled. All reusable field material was decontaminated between sites with 2% Virkon S (Antec—DuPont, Suffolk, UK) as a biosafety precaution and to avoid potential DNA cross-contamination^78^. For all filters (3 ponds × 21 months minus 1 dry pond = 62 in total), the remaining water inside was expelled until the capsule was dry, after which they were capped at both ends and immediately stored at − 21 °C in a BlueLine box (delta T, Fernwald, Germany) for transportation to the lab for further storage and analyses.

### Laboratory protocol

Prior to PCR, all eDNA samples were stored and processed in a PCR-free building dedicated to low copy number template extractions, with controlled DNA-free high-efficiency particulate air (HEPA)-filtered compartments with positive pressure to prevent eDNA sample contamination. In the first step of the extraction, 10 mL of a lysis buffer including a known concentration of IPC (KWR Watercycle Research Institute, Nieuwegein, Netherlands) (898 µL ATL buffer, 2 µL IPC, 100 µL proteinase K) was added via the inlet of each filter. The filters were subsequently recapped and placed in a rotating incubator overnight set at 56 °C. This IPC consisted of a plasmid with a 149 bp insert sequence from Dengue virus type 2 (GenBank M29095.1) and was quantified with the primers and probe shown in Table 1 (NEN 6254, 2012) in duplex with the primer/probe assays used for bullfrog detection. Preliminary analyses indicated no interference between IPC and target assays when duplexed, and similar effects of PCR inhibiting compounds (Pearson’s r = 0.48, *P* < 0.001). Comparing the concentration of IPC initially added to each filter and finally quantified by ddPCR therefore allows to standardize variation in bullfrog eDNA concentrations attributable to sample-specific differences in DNA extraction or amplification efficiency and hence to increase the comparability of eDNA samples in space and time^30,38,66^. The DNA was extracted from the filters using Qiagen’s DNeasy Blood & Tissue Kit according to manufacturer’s instructions (NatureMetrics, Surrey, England), and was finally eluted in 100 µL Tris–EDTA (10 mM Tris–HCl, 1 mM EDTA, pH 8.0) preheated at 70 °C^79^. DNA extracts obtained from the monthly sampled ponds were additionally purified with the DNeasy PowerClean Cleanup Kit (Qiagen) according to the guidelines provided by the manufacturer, and were eluted in 100 µL of TE. eDNA quantification with digital droplet PCR (ddPCR) is described in the Supplementary Methods.

### Data analysis

The total number of bullfrog DNA copies per liter filtered water (*C*_x_) was calculated for each sample using the following formula^38^:

$${\text{C}}_{{\text{x}}} = \frac{{\left( {\frac{{{\text{C}}_{{{\text{IPC}}\;{\text{initial}}}} }}{{{\text{C}}_{{{\text{IPC}}\;{\text{ observed}}}} }}} \right) \times {\text{C}}_{{\text{obs }}} \times \left( {\frac{{{\text{V}}_{{{\text{PCR}}}} }}{{{\text{V}}_{{\text{r}}} }}} \right) \times {\text{V}}_{{\text{e}}} }}{{{\text{V}}_{{\text{w}}} }}$$

where *C*_IPC initial_ is the concentration of IPC initially included in each sample (on average 1099 ± 55 copies per µL), *C*_IPC observed_ is the obtained sample-specific IPC concentration in each ddPCR reaction, *C*_obs_ is the obtained sample-specific bullfrog eDNA concentration in each ddPCR reaction (in copies per µL) adjusted for 10% loss during droplet generation, *V*_PCR_ is the total ddPCR reaction volume (in µL), *V*_r_ is the volume of the eluted extract undergoing ddPCR (in µL), *V*_e_ is the total elution volume after DNA extraction (in µL), and *V*_w_ is the total volume of filtered water (in L) (for more details see Supplementary Methods and Brys et al*.*^38^).

To quantify and compare the sensitivity of both primer/probe assays, the mean bullfrog eDNA concentration obtained per assay was calculated for the three ddPCR replicates run on each of the 25 positive field samples. Relative performance per sample was then determined based on the assay retrieving the highest concentration for that sample. The average relative performance across all samples was compared among assays using a paired two-tailed Student's *t*-Test. The difference in resolution for both the target and the IPC quantification was tested between both assays with a nonparametric paired two-tailed Wilcoxon Signed Rank Test. Linear regressions were used to test the relationships between eDNA concentration, on the one hand, and abundance and total biomass on the other by applying the function *lm*. Differences in per individual and per biomass eDNA concentration among life stages were analyzed with a nonparametric two-sided Mann–Whitney test. All statistical analyses were carried out using an alpha-value of 0.05 and with the package *stats* in RStudio version 4.0.2^80^.

### Ethics statement

All experimental protocols were approved by the Research Institute for Nature and Forest. The mesocosm experiments were carried out according to the Institutional and International ethical guidelines.

## Supplementary Information

Supplementary Information.

## Data Availability

The datasets generated during and analyzed during the current study are available from the corresponding author on reasonable request.
