# Supplementary material for: Accurate detection and quantification of seasonal abundance of American bullfrog (Lithobates catesbeianus) using ddPCR eDNA assays
Source: Sci Rep. 2021 May 28;11:11282. doi: 10.1038/s41598-021-90771-w (PMC8163797; doi:10.1038/s41598-021-90771-w)
Supplement: Supplementary file 1 — Supplementary Information. [file 41598_2021_90771_MOESM1_ESM.docx]

**Accurate detection and quantification of seasonal abundance of American bullfrog (*Lithobates catesbeianus*) using ddPCR eDNA assays**

Teun Everts^1*^, David Halfmaerten^1^, Sabrina Neyrinck^1^, Nico De Regge^1^, Hans Jacquemyn^2^, Rein Brys^1*^

^1^ Research Institute for Nature and Forest, Geraardsbergen, Belgium

^2^ KU Leuven, Department of Biology, Plant Conservation and Population Biology

* Corresponding authors

E-mail: teun.everts@inbo.be (TE), [rein.brys@inbo.be](mailto:rein.brys@inbo.be) (RB)

**Supplementary Figure S1** *In silico* alignment of amplicon sequences from the most common amphibian species co-occurring with bullfrogs in Western Europe (*Lissotriton helveticus* was excluded since no 16S rRNA sequences were available) with corresponding INSDC accession numbers compared to the bullfrog reference sequence, showing mismatches to the primer and probe binding sites for (a) the Strickler, Veldhoen, and Goldberg assays targeting the cytochrome *b* gene (upper section), and (b) the Lin assay targeting the 16S rRNA gene (lower section).

**Supplementary Table S1** Subset of eDNA samples originating from a metabarcoding study in Belgium outside the distribution range of bullfrogs using the vertebrate-specific Riaz generalist primer set (unpublished results), used for the *in situ* evaluation of the specificity of both assays under comparison. The proportional number of reads for native amphibians most commonly co-occurring with bullfrogs in Western Europe are given for thirteen ponds and the amphibian species with the highest proportional number of reads per pond are indicated in bold.

**Supplementary Figure S1**


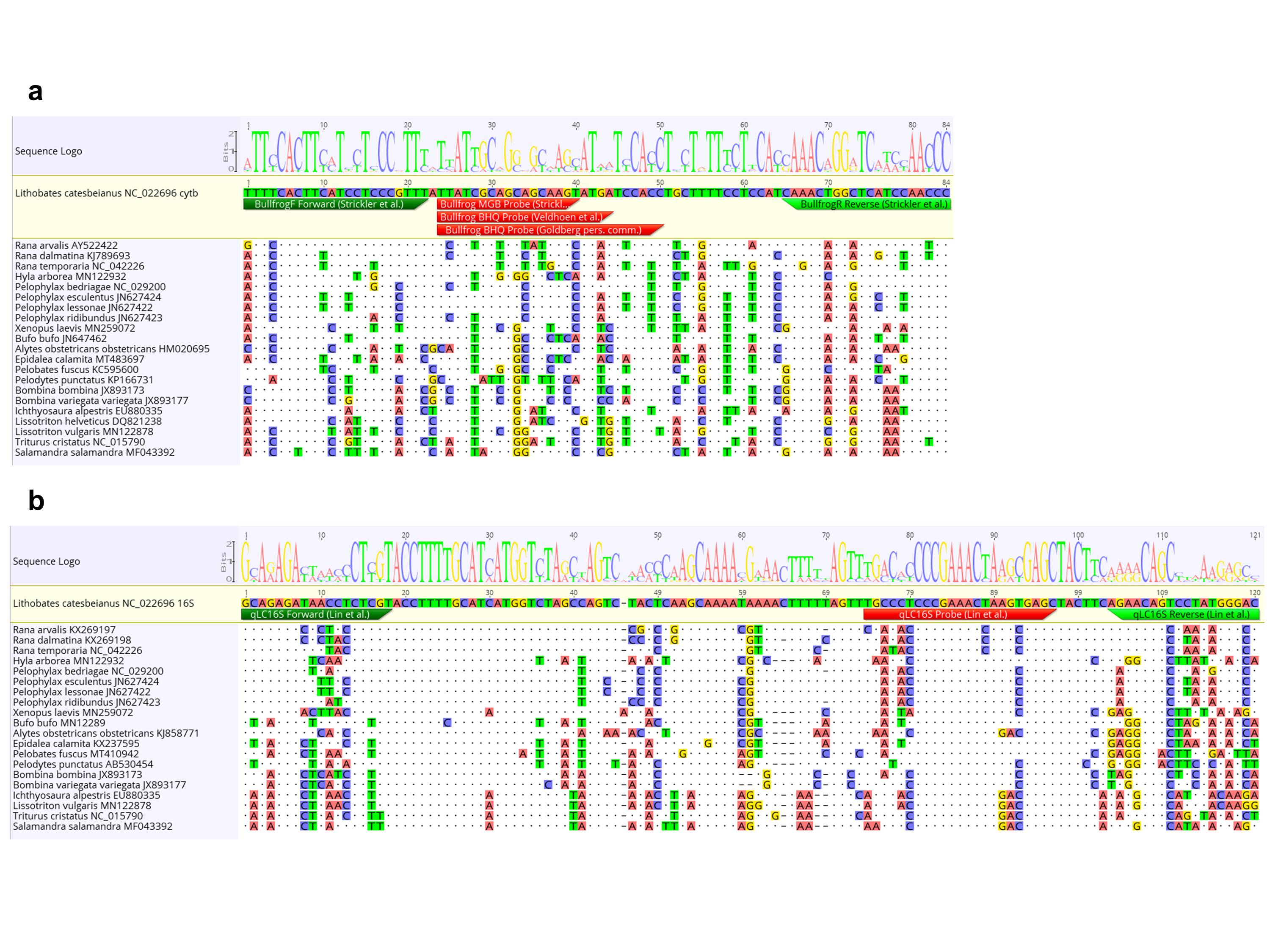


**Supplementary Table S1**

| **Amphibian**  **species**  **Pond ID** | *Ichthyosaura alpestris* | *Hyla arborea* | *Rana temporaria* | *Bufo bufo* | *Pelophylax esculenta complex* | *Rana arvalis* | *Triturus cristatus* | *Lissotriton vulgaris* | *Pelobates fuscus* | *Pelophylax bedriagae* | *Epidalea calamita* | *Lissotriton helveticus* | *Alytes obstetricans* |
| --- | --- | --- | --- | --- | --- | --- | --- | --- | --- | --- | --- | --- | --- |
| **Huldenberg 1a** | 2.045 x10^-6^ | 0 | 2.045x10^-6^ | 0 | 0.14 | 0 | 0 | 0.062 | 0 | **0.32** | 4.091x10^-6^ | 0 | **0.48** |
| **Huldenberg 5b** | 0.066 | 0 | **0.44** | 0 | 7.24x10^-5^ | 0 | 0 | **0.38** | 0 | 0.0011 | 1.20x10^-5^ | 8.39x10^-6^ | 0.10 |
| **Huldenberg 1a** | **0.68** | 0 | 0.0026 | **0.17** | 0.0058 | 0 | 0 | 0.039 | 0 | 0.043 | 1.24x10^-6^ | 0 | 0.047 |
| **Hoge Kempen 3b** | 0 | 0 | 0.048 | 3.43x10^-7^ | 0.0099 | **0.35** | 0 | 0 | 0 | 0 | 0.18 | **0.41** | 0 |
| **Hoge Kempen 3b** | 0 | 0.00017 | 0.00074 | 6.33 x10^-5^ | 0 | **0.46** | 0 | 0 | 0 | 0 | **0.20** | **0.33** | 0 |
| **Midden Limburg 4a** | 0 | 0.092 | 0.16 | 0.078 | 0.09 | **0.38** | 0.022 | 0 | 0.16 | 0 | 0 | 0.311x10^-5^ | 0 |
| **De Brand 2a** | **0.40** | 0.024 | 0 | 0 | 0.12 | 0 | 0 | **0.43** | 0 | 0 | 0 | 8.96x10^-6^ | 0 |
| **Peer** | 0.16 | 0 | 2.39x10^-6^ | 9.22 x10^-7^ | **0.53** | 7.51x10^-7^ | **0.25** | 0.013 | 0.022 | 0 | 0.016 | 8.94x10^-7^ | 0 |
| **Waasland-haven** | 0 | 0 | 0 | 0 | **0.46** | 0 | 0 | **0.53** | 0 | 0 | 0 | 0 | 0 |
| **Peer** | 0.19 | 2.94 x10^-6^ | **0.25** | **0.48** | 0 | 0 | 0.054 | 0.013 | 0 | 0 | 3.22x10^-6^ | 0 | 0 |
| **Midden Limburg 4b** | 0 | 0.016 | 0.028 | 1.24 x10^-6^ | **0.65** | 0.17 | 1.24 x10^-6^ | 0 | 0.13 | 0 | 0 | 0 | 0 |
| **De Brand 2a** | 0.080 | **0.41** | 0 | **0.34** | 1.10x10^-6^ | 0 | 0.013 | 0.15 | 0 | 0 | 2.41x10^-6^ | 0 | 0 |
| **De Brand 2b-2c** | 0.00018 | **0.50** | **0.46** | 0.00055 | 0.00040 | 0.017 | 0.00037 | 0.011 | 0 | 0.00025 | 0 | 0 | 0 |

**Supplementary Methods**

## ddPCR analyses

Digital droplet PCR (ddPCR) relies on the partitioning of DNA samples into thousands of extremely small water-in-oil droplets, each containing only one or a few copies of target DNA. Individual endpoint PCR reactions are carried out simultaneously within each droplet, which is scored as either target-positive or target-negative using probe-based fluorescence measurements (*i.e.,* a digital read-out). Poisson statistics allow the absolute quantification of the initial number of target sequences from the number of positive and negative partitions. The advantages of ddPCR compared to qPCR are fourfold: (i) the construction of calibration curves can be circumvented, (ii) a high number of individual partitions assures very precise results, (iii) the accurate detection of few target DNA particles in a background of abundant non-target DNA, and (iv) a higher resilience against PCR-inhibiting agents.

ddPCR was conducted using a QX200 ddPCR system (Bio-Rad, Temse, Belgium) in 20 μL. ddPCR Each sample included 10 μL Bio-Rad ddPCR supermix for probes (no dUTP), 750 nM of each primer, 375 nM of each probe (IDT, Leuven, Belgium), 4 μL template DNA, and was adjusted to the final volume of 20 µL by adding DEPC water (Sigma-Aldrich, Overijse, Belgium). Additionally, each ddPCR plate also included six no template controls (NTC) containing 0.1x IDTE buffer (pH 8), as well as two positive reference samples comprising DNA extracted from bullfrog tissue. After droplet generation, the ddPCR 96-well plate (Bio-Rad, cat no. 12001925) was sealed with pierceable foil (Bio-Rad, cat no. 181-4040) and brought into a C1000 Touch^TM^ Thermal Cycler with a 96-well Deep Reaction Module (Bio-Rad). PCR conditions were 10 min at 95 °C, followed by 40 cycles of denaturation for 30 sec at 94 °C and annealing/extension at optimal temperatures (60 °C for the Goldberg assay and 55°C for the Lin assay) for 60 sec, with a ramp rate of 2 °C s^-1^, followed by 10 min at 98 °C and a hold at 12 °C. ddPCR analyses of the mesocosm samples (6 technical replicates, 2x 1:2, 1:5, and 1:10 diluted) and seasonal samples (in triplicate 1:2 diluted) were run using the Goldberg assay (since this appeared to be the most sensitive and robust assay, see Results section).

Following amplification, all samples were incorporated into a QX200 droplet reader (Bio-Rad) to visualize the total amount of target-positive and target-negative droplets. Quantification of initial eDNA target copy numbers per µL was performed with the QuantaSoft software (v.1.7.4, Bio-Rad) and was estimated from the fraction of positive end-point reactions using Poisson statistics. Fluorescent thresholds for positive signals were determined according to QuantaSoft software instructions, and all droplets beyond this fluorescence threshold (Amplitude > 3000) were counted as positive events, and those below it as negative events.
